# DNA damage response and repair in perspective: *Aedes aegypti*, *Drosophila melanogaster* and *Homo sapiens*

**DOI:** 10.1186/s13071-019-3792-1

**Published:** 2019-11-11

**Authors:** Maria Beatriz S. Mota, Marcelo Alex Carvalho, Alvaro N. A. Monteiro, Rafael D. Mesquita

**Affiliations:** 1 Departamento de Bioquímica, Instituto de Química, Universidade Federal do Rio de Janeiro, Rio de Janeiro, RJ Brazil; 2 Instituto Federal do Rio de Janeiro, Rio de Janeiro, RJ Brazil; 3 Instituto Nacional de Câncer, Divisão de Pesquisa Clínica, Rio de Janeiro, RJ Brazil; 4 Cancer Epidemiology Program, H. Lee Moffitt Cancer Center and Research Institute, Tampa, FL USA; 5 Instituto Nacional de Ciência e Tecnologia em Entomologia Molecular, Universidade Federal do Rio de Janeiro, Rio de Janeiro, RJ Brazil

**Keywords:** *Aedes aegypti*, DDR, DNA damage response, DNA repair

## Abstract

**Background:**

The maintenance of genomic integrity is the responsibility of a complex network, denominated the DNA damage response (DDR), which controls the lesion detection and DNA repair. The main repair pathways are base excision repair (BER), nucleotide excision repair (NER), mismatch repair (MMR), homologous recombination repair (HR) and non-homologous end joining repair (NHEJ). They correct double-strand breaks (DSB), single-strand breaks, mismatches and others, or when the damage is quite extensive and repair insufficient, apoptosis is activated.

**Methods:**

In this study we used the BLAST reciprocal best-hit methodology to search for DDR orthologs proteins in *Aedes aegypti*. We also provided a comparison between *Ae. aegypti*, *D. melanogaster* and human DDR network.

**Results:**

Our analysis revealed the presence of ATR and ATM signaling, including the H2AX ortholog, in *Ae. aegypti*. Key DDR proteins (orthologs to RAD51, Ku and MRN complexes, XP-components, MutS and MutL) were also identified in this insect. Other proteins were not identified in both *Ae. aegypti* and *D. melanogaster*, including BRCA1 and its partners from BRCA1-A complex, TP53BP1, PALB2, POLk, CSA, CSB and POLβ. In humans, their absence affects DSB signaling, HR and sub-pathways of NER and BER. Seven orthologs not known in *D. melanogaster* were found in *Ae. aegypti* (RNF168, RIF1, WRN, RAD54B, RMI1, DNAPKcs, ARTEMIS).

**Conclusions:**

The presence of key DDR proteins in *Ae. aegypti* suggests that the main DDR pathways are functional in this insect, and the identification of proteins not known in *D. melanogaster* can help fill gaps in the DDR network. The mapping of the DDR network in *Ae. aegypti* can support mosquito biology studies and inform genetic manipulation approaches applied to this vector.

## Background

*Aedes aegypti* is one of the most important insect vectors due to its ability to transmit dengue, Zika, chikungunya, and yellow fever [1]. The disease vector capacity of this mosquito is related to its blood-feeding habits. In a single meal *Ae. aegypti* females can ingest an amount of blood up to three times their body weight [2, 3]. Hemoglobin, which is about 60% of the blood protein fraction, releases its prosthetic group heme when digested in mosquito gut. In the insect midgut, heme accumulation and hydrolysis by heme oxygenase lead to iron release that catalyzes the formation of reactive oxygen species (ROS) *via* Fenton reaction [2]. In larval stages, water pollutants, heavy metals and plant metabolites present in breeding sites, as well as UV exposure, contribute to ROS formation and can alter insect physiology and insecticide tolerance [4, 5].

Low levels of ROS are important for many biological processes such as signal transduction, and insect immunity [6, 7]. However, high levels of ROS can induce lipid peroxidation, protein and DNA oxidation, generate DNA single-strand breaks (SSBs) and double-strand breaks (DSBs) [8–10].

To repair DNA damage and maintain genome integrity, organisms rely on a complex system denominated the DNA damage response (DDR). The DDR includes signaling and repair pathways, as base excision repair (BER), nucleotide excision repair (NER), mismatch repair (MMR), homologous recombination repair (HR) and non-homologous end joining repair (NHEJ) [11–13]. In addition, when the damage is quite extensive and repair insufficient, apoptosis is activated [14].

The DDR network has been extensively studied in model organisms such as *Drosophila melanogaster*, that encodes many key DDR proteins [15], but little is known about these pathways in insect vectors [16]. In mosquitoes the repair of DSB has been initially studied to improve genomic manipulation and generation of transgenic insects [17–20]. Thus, the wide identification of the DDR players in *Ae. aegypti* can support the genomic manipulation tools development, and the resistance and transmission-blockage studies.

In this study we used bioinformatics tools to search for and annotate proteins from and related to DDR pathways in *Ae. aegypti*. We show here that key genes coding for DDR proteins are present, suggesting that the main DDR pathways are functional in this organism. Additionally, *Ae. aegypti*, like other dipterans, lacks important DDR proteins such as BRCA1, TP53BP1 and XRCC4, raising questions about how they deal with the lack of these DDR components.

## Databases

The DDR signaling and repair pathways analyzed were as follows: ATR signaling; double-strand break repair (DSB); homologous recombination repair (HR); non-homologous end joining repair (NHEJ); mismatch repair (MMR); base excision repair (BER); and nucleotide excision repair (NER).

The following databases were used: (i) Uniprot-Swissprot release May 2018 (http://ftp.ebi.ac.uk); (ii) one custom DDR database compiled by us containing DDR proteins from *Homo sapiens*, *Apis mellifera* and *Drosophila melanogaster*. The *H. sapiens* DDR proteins listed in Reactome pathways “base excision repair”, “nucleotide excision repair”, “mismatch repair”, “DNA double-strand break response”, “HDR through homologous recombination (HRR) or single-strand annealing”, “nonhomologous end-joining (NHEJ)”, “HDR through MMEJ (alt-NHEJ) and “DNA damage reversal” plus *A. mellifera* and *D. melanogaster* DDR proteins listed in literature (Arcas et al. [25]) were obtained from database (i); (iii) *Ae. aegypti* proteins version 5.1 from VectorBase; (iv) KEGG eukaryotes (KE) proteins, release 5 June 2017; (v) Gene Ontology (GO) proteins, release August 2018 (http://archive.geneontology.org/); and (vi) Conserved Domain Database (CDD), version 3.16 from NCBI (ftp://ftp.ncbi.nlm.nih.gov).

Reciprocal best-hit methodology [21] was used to manually identify and annotate *Ae. aegypti* orthologs for the proteins present in the DDR database (Additional file 1: Figure S1). The first BLASTP used proteins from DDR database (ii) as queries and *Ae. aegypti* database (iii) as subject. The blastp e-value cut-off (10^−15^) was determined experimentally by our group to restrict the BLAST results, lowering the potentially false positive hits. The top 5 hits were considered if the e-value was smaller than 10^−15^. These *Ae. aegypti* hit proteins were compared by BLASTP with the databases db(i), db(iv), db(v) and db(vi). The top 2 back-hits were considered if the e-value was smaller than 10^−15^. The orthology was assumed to the *Ae. aegypti* protein that have (i) both top 2 back-hits (for databases db(i), db(iv) and db(v)) with the same annotation as the initial query; and (ii) the same typical conserved domains (database db(vi) result) as those present in the initial query.

Multiple sequence alignment was carried out using the Clustal Omega web server with standard parameters [22]. Kinase-specific and unspecific phosphorylation sites prediction was made using NetPhos 3.1 web prediction server [23] also with standard parameters.

## Signal transduction

The DDR signaling pathway consists of DNA damage sensors, signal transducers and effectors proteins, and at core of this machinery are the transducer kinases ATM (ataxia telangiectasia mutated), ATR (ATM and Rad3-related) DNAPKcs (DNA-dependent protein kinase, catalytic subunit). These three phosphoinositide 3-kinase related kinases (PIKKs) are responsible to phosphorylate the effector proteins, which participate in cell cycle control, DNA repair pathways and apoptosis. ATM and DNAPKcs are involved in repair of double-strand breaks (DSBs) while ATR is activated by a variety of damages, being important in signaling of UV lesions, stalled replication forks and in damage surveillance during DNA replication [24]. ATM seems to have emerged in plants, while DNAPKcs ATR appears in early eukaryotes [25]. These kinases are present in *Ae. aegypti*, whereas *D. melanogaster* encodes orthologs only for ATM and ATR [25]. The role of ATR, ATM and DNAPKcs will be discussed in the next sections.

**Fig. 1:**
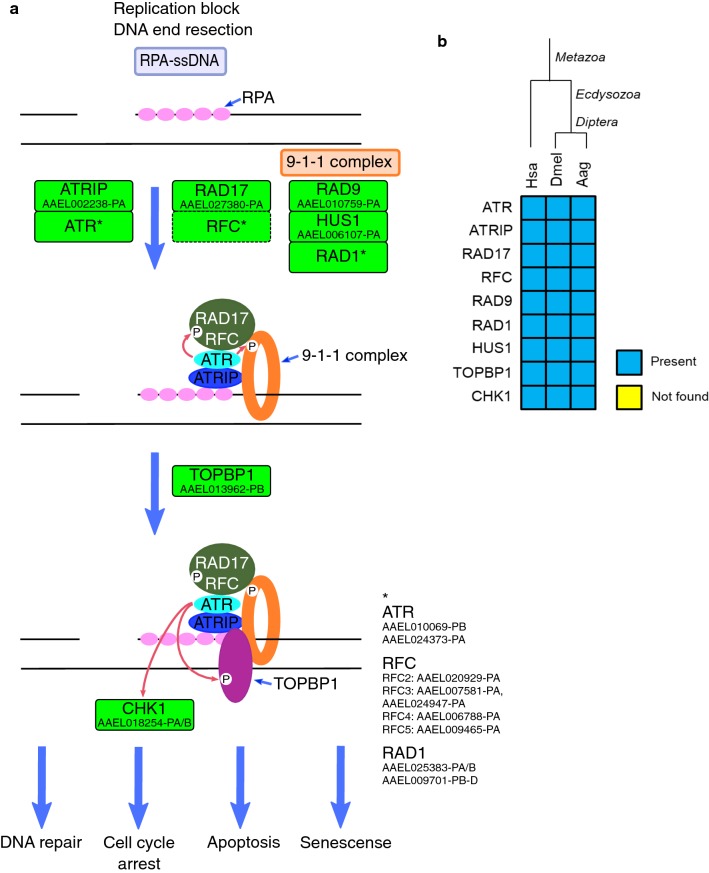
**a** ATR signaling in *Ae. aegypti*. Rectangles: green, identified; solid line, protein; dashed, protein complex. Replication fork proteins were omitted to improve clarity. **b** Heatmap of *H. sapiens* (Hsa); *D. melanogaster* (Dmel) and *Ae. aegypti* (Aag) proteins. Protein codes are provided in Additional file 2: Table S1

ATR is recruited in response to single-stranded DNA (ssDNA), through the binding of ATR-interacting protein (ATRIP) to RPA, that coats ssDNA structure [26]. RPA-ssDNA also recruits Rad17-RFC clamp loader to ssDNA/dsDNA junction, which loads RAD9-RAD1-HUS1 (9-1-1) clamp onto double-strand DNA (dsDNA) [27]. 9-9-1 promotes the recruitment of TOPBP1 that fully activates ATR, which phosphorylates effector proteins such as checkpoint kinase 1 (Chk1) involved in arrest of cell cycle progression [28, 29]. The proteins of the ATR network seem to have appeared in early eukaryotes, with exception of ATRIP that emerged in plants, and CHK1 that appeared before fungi and animals split [25]. Due to the early origin of these pathways, all proteins were identified in *Ae. aegypti* (Fig. 1). The complete list of *Ae. aegypti* ATR signaling proteins is provided in Additional file 2: Table S1.

## Double-strand break repair

Double-strand breaks (DSBs) are potential harmful lesions that can be repaired by homologous recombination (HR) and by non-homologous end joining (NHEJ) [30]. The MRN complex, composed of RAD50, NBN (also known as NBS1 and XRS2 in yeast) and MRE11, is the sensor that recognizes a DSB and recruits ATM to damage site [31]. Activated ATM phosphorylates CHK2 and p53, regulating cell cycle arrest, senescence and apoptosis in human cells [11]. ATM also phosphorylates the adjacent histones H2A/H2AX, producing gamma-H2A (γH2A) and gamma-H2AX (γH2AX), which is relevant for the foci formation and the recruitment of the mediator of DNA damage checkpoint 1 (MDC1) [32]. MDC1 promotes ATM signaling amplification and recruits E3 ubiquitin ligase RNF8 that is responsible for the initial ubiquitination of the histones H2A/H2AX followed by poly ubiquitination by E3 ubiquitin ligase RNF168 [33–36]. This ubiquitination process is necessary for the recruitment of BRCA1-A complex, composed of BRCA1-BARD1 heterodimer, RAP80, ABRAXAS, BRCC3, BRE, BABAM1 [35, 37, 38].

The choice of DSB repair pathway depends of cell cycle stage. In the S and G2, CtIP associates with BRCA1 and MRN complex to stimulate DSB end resection promoting homologous recombination (HR) [39, 40], whereas in G1 the association of TP53BP1 with RIF1 and PTIP inhibits the DSB end resection leading to NHEJ [41].

**Fig. 2:**
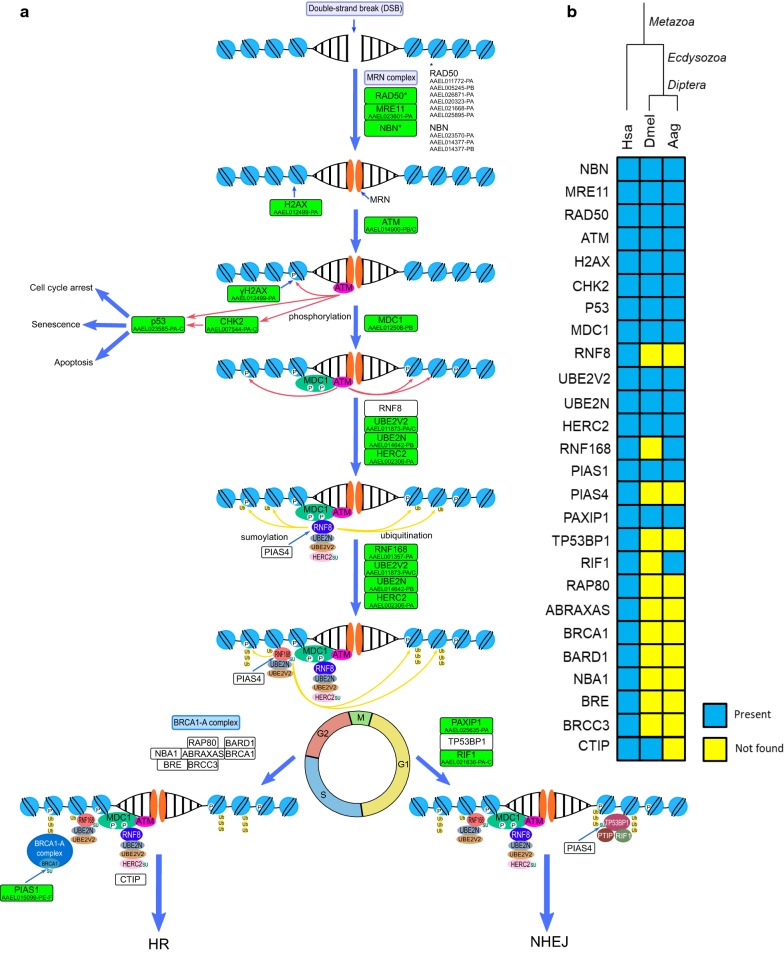
**a** Double-strand break (DSB) signaling in *Ae. aegypti*. Rectangles: green, identified; white, not identified. **b** Heatmap of *H. sapiens* (Hsa), *D. melanogaster* (Dmel) and *Ae. aegypti* (Aag) proteins. Protein codes are provided in Additional file 2: Table S2

The genes encoding proteins at the first steps of the pathway, damage recognition and initial response, possess an early origin. MRE11, RAD50, CHK2 and p53 all emerged in early eukaryotes, and NBN appeared in plants [25]. In *Ae. aegypti*, the MRN complex (NBN: AAEL023570-PA, AAEL014377-PA/B; MRE11: AAEL023601-PA; RAD50: AAEL011772-PA, AAEL005245-PB, AAEL026871-PA, AAEL020323-PA, AAEL021668-PA, AAEL025895-PA), ATM (AAEL014900-PB/C), CHK2 (AAEL007544-PA-C) and p53 (AAEL023585-PA-C) are all present (Fig. 2).

Arcas et al. [25], suggested that MDC1 appeared only in vertebrates; however, in our analysis we were able to identify an ortholog of this protein in *Ae. aegypti* (AAEL012508-PB), which is also present in *D. melanogaster* [15] (Fig. 2).

**Fig. 3:**
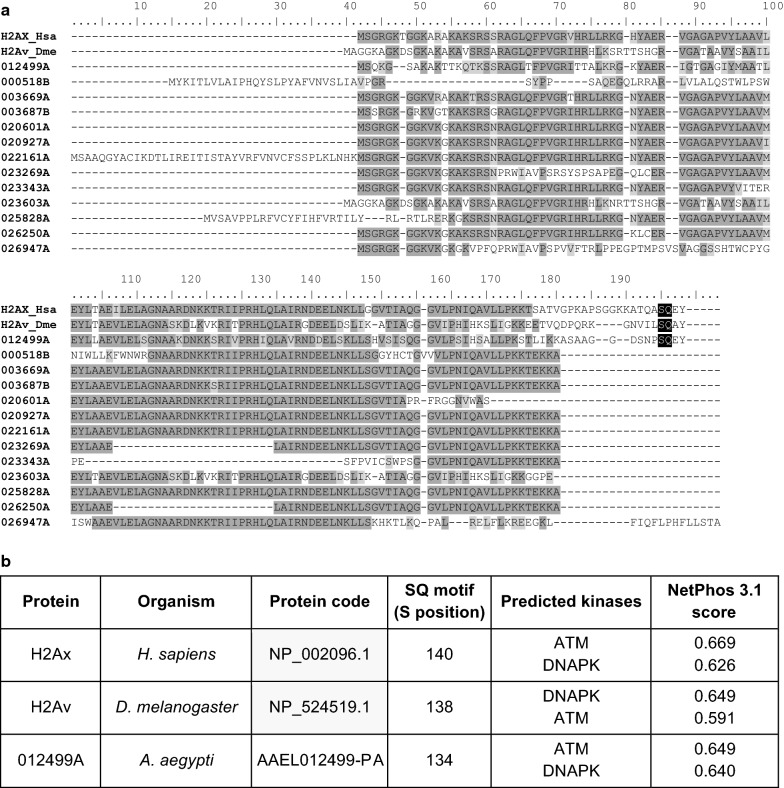
**a** Multiple sequence alignment of H2Ax histones. Identical proteins were clustered and codes were shortened for brevity. Background: light grey, low amino acid conservation; dark, high conservation; black, SQ-motif. **b** SQ-motif phosphorylation prediction. Only predictions above NetPhos cut-off score (0.5) were considered

The histone H2A is highly conserved through the evolution, being identified in early eukaryotes [25]. Some of H2A histone variants include the SQ motif, located at the C-terminal region, which is required for ATM phosphorylation. The variant H2AX, in humans, possesses the SQ motif and is phosphorylated in Ser139. In *D. melanogaster*, the H2Av is the functional ortholog of human H2AX, being phosphorylated at Ser138, in response to DSB [42]. We identified 94 histones H2A in *Ae. aegypti* and, to search for the SQ motif, we made an alignment between human H2Ax, *D. melanogaster* H2Av, and H2A identified in *Ae. aegypti* (Fig. 3a). The C-terminal region of AAEL012499-PA was the only one among all the *Ae. aegypti* histones that showed the SQ motif. Furthermore, it aligned correctly with the SQ motifs from the human H2AX and *D. melanogaster* H2Av. Phosphorylation prediction analyses, made with NetPhos 3.1 web prediction server, also showed the same result to AAEL012499-PA Ser134, to H2AX Ser140 (NCBI and Uniprot records to human H2AX (NP_002096.1) point Ser140 as the phosphorylation position but the literature [42] points Ser139, despite the numbering differences both refer to the same serine) and to H2Av Ser138, indicating the SQ motif is phosphorylated by ATM (Fig. 3b). Taken together, these data suggest that AAEL012499-PA is the *Ae. aegypti* functional ortholog of the human H2AX and *D. melanogaster* H2Av. The H2A identified in *Ae. aegypti* are provided in Additional file 2: Table S3.

The ubiquitin-protein ligase RNF8 was detected in early eukaryotes, while the RNF168 was identified only in Chordata. Both are absent in *D. melanogaster* and in the model species *C. elegans* and *S. cerevisiae* [25]. *Aedes aegypti* lacks the RNF8 ubiquitin ligase, but, curiously, encodes an ortholog for the RNF168 (AAEL001357-PA) (Fig. 2).

The SUMO E3 ligase PIAS4 is absent in *Ae. aegypti*, but PIAS1 (AAEL015099-PE/F) is present (Fig. 2). In vertebrates both proteins are involved in sumoylation of DSB response/repair proteins, such as HERC2, RNF168, BRCA1 and TP53BP1 [43–45]. While PIAS4 is present only in vertebrates, PIAS1 appeared earlier, before division of plants [25], suggesting that PIAS1 should plays the role of PIAS4, not only in *Ae. aegypti*, but also in other species that lack this protein, such as *D. melanogaster*.

The CtIP and the BRCA1-A complex, including BRCA1, were not identified in *Ae. aegypti* (Fig. 2). CtIP emerged in Bilateria and an ortholog have already been found in *D. melanogaster* [15, 25]. Most BRCA1-A complex proteins possess an early origin; BRCA1, BRE and BRCC3, originated in early eukaryotes, while NBA1 and BARD1 in the common ancestor of plants and animals. Only RAP80 appeared in vertebrates. However, this complex is also absent in *D. melanogaster* and seems to have been lost in Diptera [25]. It has already been reported that the components of DSB response have originated in different periods of time, suggesting that this pathway may have assembled in a modular way during evolution [25]. The lack of BRCA1-A complex proteins together with the fact that HR is functional [46–48] suggest that dipterans should have rewired the HR pathway activation.

TP53BP1 originated in Metazoa, whereas PAXIP1 and RIF1 emerged in early eukaryotes [25]. Although TP53BP1 is absent in *Ae. aegypti*, both PAXIP1 and RIF1 are present. Regarding the TP53BP1 absence, it has already been shown that PAXIP1 could also associate with ARTEMIS (a known NHEJ factor detailed below), induced by DNA damage. This association happens downstream of TP53BP1 and leads to the trimming of the DNA ends to facilitate NHEJ and avoid the extensive resection necessary for HR [49]. As ARTEMIS is a conserved nuclease that is present in *Ae. aegypti*, it is a possibility that in this insect the interaction between ARTEMIS and PAXIP bypass TP53BP1 signaling and promotes NHEJ activation. The complete list of *Ae. aegypti* double-strand break repair proteins is provided in Additional file 2: Table S2.

### Homologous recombination

DSB repair by homologous recombination (HR) occurs during S and G2 phases of the cell cycle and uses the sister chromatid as a template for repair [50].

**Fig. 4:**
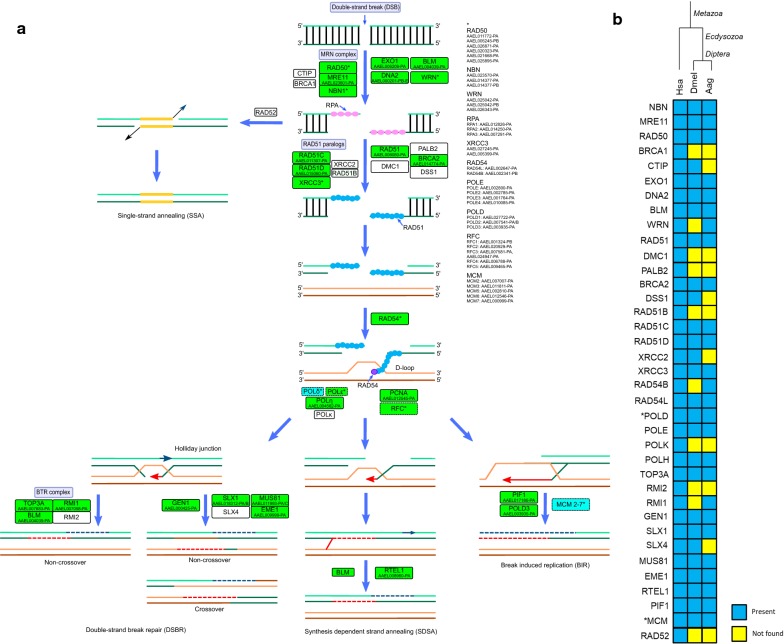
**a** Homologous recombination (HR) repair in *Ae. aegypti*. Rectangles: solid line, protein; dashed, protein complex; green, identified; cyan, partially identified; white, not identified. **b** Heatmap of *H. sapiens* (Hsa), *D. melanogaster* (Dmel) and *Ae. aegypti* (Aag) proteins. Protein codes are provided in Additional file 2: Table S4. *Subunits not identified: Aag-MCM4, Aag-POLD4 and Dmel-POLD4

To initiate, HR requires the resection of the DSB, which is initially carried out by the association of the MRN complex, CtIP and BRCA1, followed by the exonuclease EXO1 or the endonuclease DNA2, in a process facilitated by the helicases BLM and WRN [40, 51–54]. As discussed above, orthologs of human CtIP and BRCA1 were not found in *Ae. aegypti*, being still unclear how this organism carries out this step. The helicase BRIP1, that interacts with BRCA1 and is supposed to participate in the recruitment of RPA and RAD51 [55], is also absent. The MRN complex as well as the three RPA subunits were identified in *Ae. aegypti* (discussed above). EXO1, DNA2 and WRN emerged in early eukaryotes, and BLM is an ancient protein found in prokaryotes. All these proteins are present in *Ae. aegypti* (Fig. 4).

The formation of invasive RAD51 nucleoprotein filaments occurs by the replacement of RPA from ssDNA by RAD51, mediated by BRCA2 and PALB2 [56, 57]. The filaments are stabilized by RAD51 paralogs and invade the sister chromatid in cooperation with RAD54 [58, 59]. After invasion, the replicative DNA polymerases (POL δ, ε) or translesion DNA polymerases (POL η, κ) extend the DNA strand generating a D-loop [60]. The RAD51 allow the HR during meiosis and mitosis. It is one of the two eukaryotic functional homologs of the strand exchange bacterial RecA [61]. This protein is present in early eukaryotes [25] and an ortholog was also found in *Ae. aegypti* (AAEL006080-PA). The other eukaryotic RecA functional homolog is DMC1, which acts only in meiosis [61]. An ortholog of this protein was not identified in *Ae. aegypti* and is also lacking in *D. melanogaster*. Humans encode five RAD51 paralogs: RAD51B, RAD51C, RAD51D, XRCC2 and XRCC3 (Fig. 4). In *Ae. aegypti* RAD51B was not identified, as expected since it is lacking in Ecdysozoa [15]. Surprisingly, XRCC2 that is present in *D. melanogaster* [15] and in other mosquitoes such as *An. gambiae* and *Cx. quinquefasciatus* (KEGG orthology group K10879) was not found. Otherwise, RAD51C (AAEL011307-PA), RAD51D (AAEL015060-PA) and XRCC3 (AAEL027245-PA, AAEL005399-PA) were all identified in *Ae. aegypti*. BRCA2 emerged in early eukaryotes [25] and was identified, but PALB2 (Fig. 4), which seems to have appeared in vertebrates [25], was not. Two orthologs of RAD54 were identified in *Ae. aegypti*, RAD54L and RAD54B, otherwise one of them (RAD54B) have been lost in many insects, including *D. melanogaster* [15]. The replicative DNA polymerases POL δ and ε are complexes both formed by four subunits. It was not found in, *Ae. aegypti*, the subunit 4 of POL δ but the catalytic subunit was (POLD1 - AAEL027722-PA; POLD2 - AAEL007541-PB, AAEL007541-PA; POLD3 - AAEL003935-PA). All the subunits of POL ε (POLE - AAEL002800-PA; POLE2 - AAEL002785-PA; POLE3 - AAEL001764-PA; POLE4 - AAEL010085-PA) were found. The translesion DNA polymerase POL η (AAEL004562-PA) was identified in *Ae. aegypti*, but the POL κ was not, it is also absent in *D. melanogaster* and in several insects [15] (Fig. 4).

D-loop structures can be solved by three pathways: double-strand break repair (DSBR), synthesis-dependent strand annealing (SDSA) or break induced replication (BIR) [50]. In the DSBR the D-loop is processed by the formation of the Holliday junction that can be dissolved (by the BTR complex generating non-crossover products) or resolved (by the nucleases SLX1-SLX4 and MUS81-EME1 or GEN1 forming both crossover and non-crossover products) [62–65]. The BTR complex proteins TOP3A and BLM are ancient proteins, being found even in prokaryotes, whereas RMI1 and RMI2 emerged in plants and animals, respectively. Both MUS81 and SLX1, as well as GEN1, seems to have originated in early eukaryotes, EME1 and SLX4 emerged later in animals [25]. Of these proteins, only RMI2 and SLX4 were not identified in *Ae. aegypti.* RMI2 is also lacking in *D. melanogaster* and in most insects [15]. Although it was proposed that dipterans have lost RMI1 [15], we identified an ortholog of this protein in *Ae. aegypti* (Fig. 4).

In the SDSA, the invading strand dissociates from the sister chromatid and anneals with the complementary strand of the broken DNA end, which results in non-crossover products [50]. This pathway is carried out by the helicases RTEL1 or BLM [66, 67], which were both identified in *Ae. aegypti* (RTEL1 - AAEL008960-PA; BLM - AAEL004039-PA) (Fig. 4).

In BIR a replication fork is assembled after D-loop formation and the entire chromosome arm is synthesized [68]. BIR was extensively studied in yeasts and is carried out by Pol 32 (POLD3 ortholog) and the helicases PIF1 and MCM2-7 [69, 70]. All these proteins, except for MCM4, are found in *Ae. aegypti* (POLD3; PIF1 - AAEL017186-PA; MCM2 - AAEL007007-PA; MCM3 - AAEL011811-PA; MCM5 - AAEL002810-PA; MCM6 - AAEL012546-PA; MCM7 - AAEL000999-PA).

The DSB can also be repaired by the RAD51 independent pathway, denominated single-strand annealing (SSA), which anneals complementary DSB ends generated by the extensive resection, in a process mediated by RAD52 [71], that is absent in *Ae. aegypti* (Fig. 4). In *D. melanogaster* SSA occurs normally although the absence of RAD52. The lack of RAD52 and BRCA1 or PALB2 is lethal to human cells [72], questions about how dipterans deal with the loss of these proteins, have already been raised, but the answer is still unknown [15]. The complete list of *Ae. aegypti* HR proteins is provided in Additional file 2: Table S4.

### Non-homologous end joining (NHEJ)

NHEJ is responsible for re-joining DNA broken ends and is the main DSB repair pathway in eukaryotes, occurring in G1 phase [73].

**Fig. 5:**
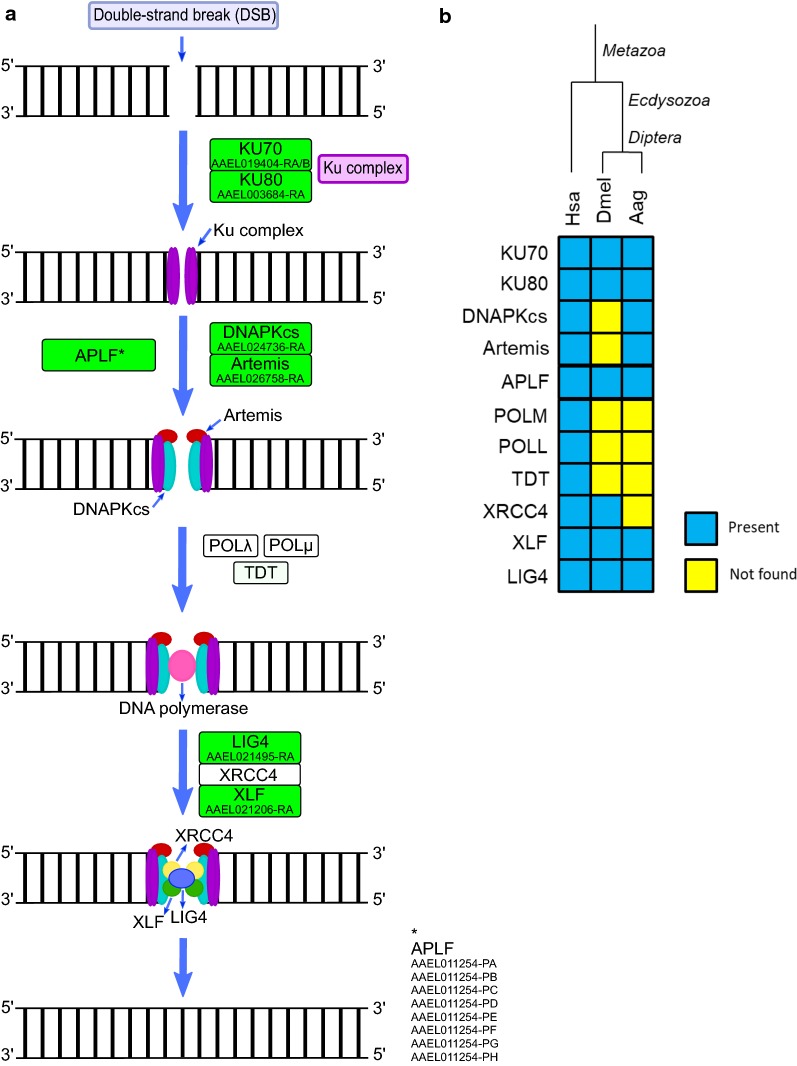
**a** Non-homologous end joining (NHEJ) repair in *Ae. aegypti*. Rectangles: green, identified; white, not identified. **b** Heatmap of *H. sapiens* (Hsa), *D. melanogaster* (Dmel) and *Ae. aegypti* (Aag) proteins. Protein codes are provided in Additional file 2: Table S5

The first step of NHEJ is the binding of Ku complex (KU70 and KU80) to DSBs, which prevents the DNA ends resection and recruits the DNA-dependent protein kinase catalytic subunit (DNAPKcs), forming a multiprotein complex in both DSB ends that interact and aligns the broken ends [74–77]. KU70, KU80 and DNAPKcs were originated in early eukaryotes, being identified in *Ae. aegypti*. Curiously, DNAPKcs is lacking in *D. melanogaster* and in many insects [15] (Fig. 5).

Subsequently, when the overhangs are not complementary, DNAPKcs activates ARTEMIS, an endonuclease that processes the broken ends to find cohesive nucleotides [78]. ARTEMIS is lacking in *D. melanogaster* and is suggested to be lost in dipterans [15]. However, we could identify an ortholog of this protein (AAEL026758-PA) in *Ae. aegypti* (Fig. 5). Although ARTEMIS is the major nuclease in NHEJ, there are others proteins that might be involved in DSBs end resection such as the PNKP-like factor (APLF), polynucleotide kinase/phosphatase (PNKP), aprataxin (APTX), tyrosyl DNA phosphodiesterase 1 (TDP1) and tyrosyl DNA phosphodiesterase 2 (TDP2) [79–81]. All these proteins were found in *Ae. aegypti* (APLF - AAEL011254-PA-H; APTX - AAEL014945-PB-D; PNKP - AAEL025882-PA; TDP1 - AAEL011629-PB-C), except for TDP2, which is also lacking in *D. melanogaster* and *A. mellifera* [25].

The processing of DNA ends continues with the Pol X family polymerases that fill small single-strand gaps in DSB ends. The POL μ and POL λ, are members of this family, and both can incorporate nucleotides in a template-dependent and independent manner [79, 82–84]. *Aedes aegypti* lacks the polymerases from Pol X family (Fig. 5) suggesting that this step occurs without the dNTPs insertion; however, other polymerases can incorporate dNTPs in a template-dependent manner during NHEJ [79]. The Pol X family polymerases are also absent in most insects, including *D. melanogaster* [15].

In the last step, the non-homologous end-joining factor 1 (XLF) interacts with the XRCC4-LIG4 complex (X-ray repair cross-complementing protein 4 - DNA ligase 4), to catalyze the DSB ligation [85, 86]. Both LIG4 (AAEL021495-PA) and XLF (AAEL021206-PA) were found in *Ae. aegypti* (Fig. 5). XRCC4 was not found in this insect (Fig. 5), although it is present in some Diptera, such as *D. melanogaster*, and its emergence reported before plants [15, 25, 87]. Further investigation is necessary to know if (and how) NHEJ works in mosquitoes without XRCC4 as it seems to be very important. The knockout of XRCC4 in mouse cells results in 20-fold reduction of NHEJ, increasing the ends degradation and ends joining by microhomology [88]. The complete list of *Ae. aegypti* NHEJ proteins is provided in Additional file 2: Table S5.

### Microhomology-mediated end joining (MMEJ)

The DSB end joining can also occur by microhomology-mediated end joining (MMEJ), also known as alternative NHEJ (alt-NHEJ), which does not require ATM activation or classical NHEJ components such as the Ku complex and XRCC4-LIG4 [89]. To initiate, MMEJ needs a limited end resection that, as in HR, is mediated by MRN complex and CtIP [90]. The ssDNA overhangs recruit PARP1 or PARP2, POLθ and 5-flap endonuclease (FEN1) involved in the microhomology events, with POLθ responsible to promote the 3′-ssDNA overhangs annealing [91, 92]. Finally, the MRN complex recruits XRCC1 and LIG3 to catalyze DNA ends ligate [93].

**Fig. 6:**
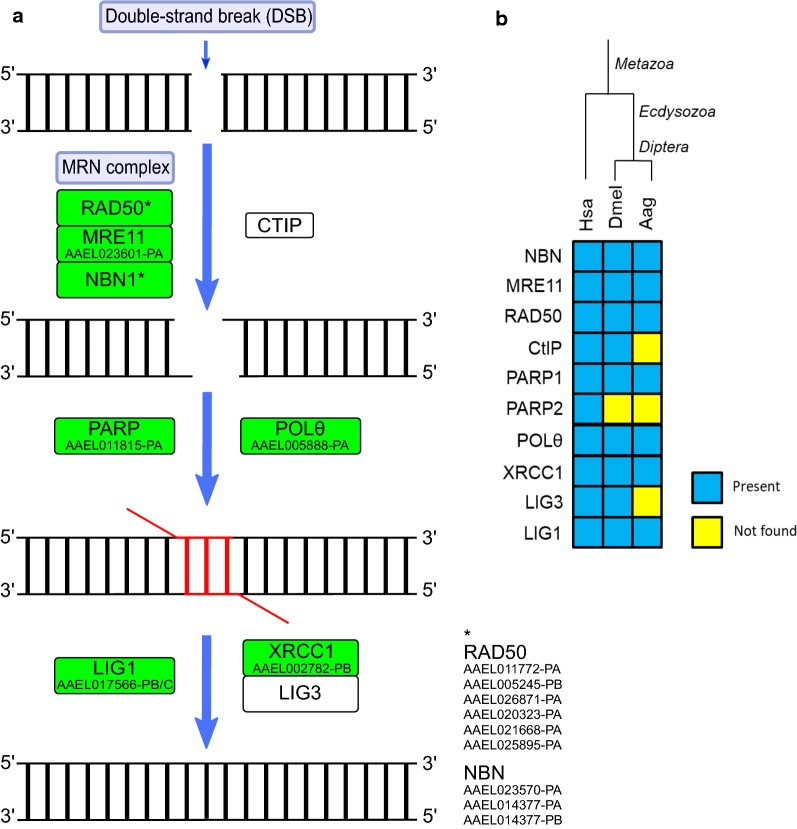
**a** Microhomology-mediated end joining (MMEJ) repair in *Ae. aegypti*. Rectangles: green, identified; white, not identified. **b** Heatmap of *H. sapiens* (Hsa), *D. melanogaster* (Dmel) and *Ae. aegypti* (Aag) proteins. Protein codes are provided in Additional file 2: Table S6

As already discussed, CtIP was not identified in *Ae. aegypti*, although *D. melanogaster* encodes a functional ortholog of this protein. Orthologs for PARP1, FEN1 and XRCC1 were found in *Ae. aegypti*, but LIG3 is absent in this insect. As these proteins participate in BER, they will be discussed in more detail later in this paper. Furthermore, an ortholog of POLθ (AAEL005888-RA) was found in *Ae. aegypti* and is also present in *D. melanogaster* (Fig. 6). In fact, the role of POLθ in MMEJ was first identified in this fly, during a P-element transposition experiment [94]. The presence of the proteins involved in MMEJ, especially POLθ, in *Ae. aegypti* suggests that this pathway is functional in this mosquito. The absence of LIG3 may not affect MMEJ in *Ae. aegypti* due to the possible role of LIG1 in this process [95]. The complete list of *Ae. aegypti* MMEJ proteins is provided in Additional file 2: Table S6.

## Mismatch repair

DNA mismatch repair (MMR) is a highly conserved pathway responsible for recognizing and correcting mismatched base pairs and insertion/deletion loops (IDLs), that occur mostly during the replication process [96].

In prokaryotes, the MutL and MutS proteins are the central players of MMR [97]. In eukaryotes, MutS homologs form the functional heterodimers MutSα (MSH2-MSH6) and MutSβ (MSH2-MSH3), that repair mismatches and IDLs of up to two bases and of more than two bases, respectively [98]. Eukaryote MutL homologs form three functional heterodimers: MutLα (MLH1-PMS2), MutLβ (MLH1-MLH3) and MutLγ (MLH1-PMS1); however, MutLα is the one most involved in MMR [99–101].

**Fig. 7:**
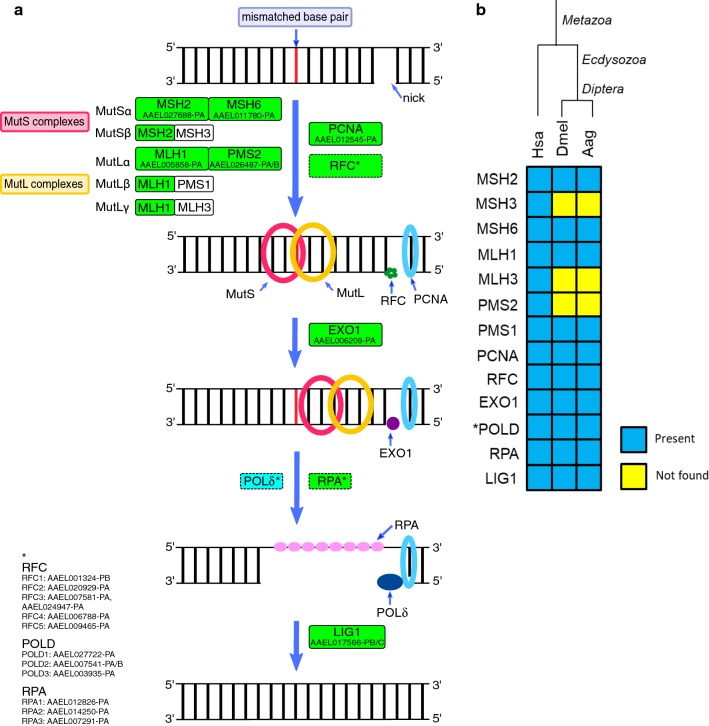
**a** Mismatch repair (MMR) repair in *Ae. aegypti*. Rectangles: solid line, protein; dashed, protein complex; green, identified; cyan, partially identified; white not identified. **b** Heatmap of *H. sapiens* (Hsa), *D. melanogaster* (Dmel) and *Ae. aegypti* (Aag) proteins. Protein codes are provided in Additional file 2: Table S7. *Subunits not identified: Aag-POLD4 and Dmel-POLD4

MutS and MutL homologs possess an early origin, since MSH3 and MSH6 and MLH1 are ancient proteins, being present in prokaryotes, and MSH2, PMS2 and MLH3 (KEGG orthology group K08739) emerged in early eukaryotes [25]. Only PMS1 appears later in Metazoa (KEGG orthology group K10864). The MSH2 (AAEL027688-PA), MSH6 (AAEL011780-PA), MLH1 (AAEL005858-PA) and PMS2 (AAEL026487-PA/B) were found in *Ae. aegypti*, while the MSH3, MLH3 and PMS1 were not (Fig. 7). They seem to have been lost in the species of Diptera [15]. The absence of these proteins suggests that dipterans should not be able to form the MutSβ, MutLβ and MutLγ complexes, at least as vertebrates do, and raises questions about how they deal with mismatches and if MutSα and MutLα are enough to do this task.

Before excision, the 5′-ends of Okazaki fragments and PCNA help discriminate between the leader and the lagging strand [102, 103]. Subsequently, the excision is orchestrated by EXO1 in cooperation with PCNA [104, 105], POL δ synthesizes a new fragment and DNA ligase I (LIG1) catalyzes strand ligation [106, 107].

The PCNA and LIG1 proteins, present in prokaryotes and eukaryotes, were both found in *Ae. aegypti*. As discussed above, RPA, EXO1 and POL δ (expect subunit 4) were all identified in this mosquito (Fig. 7). The complete list of *Ae. aegypti* MMR proteins is provided in Additional file 2: Table S7.

## Base excision repair (BER) and single-strand break repair (SSBR)

Base excision repair (BER) is responsible to handle with endogenous small base lesions as oxidation, alkylation, deamination and depurination. This pathway also repairs abasic sites (AP sites) and single-strand breaks (SSBs) [108, 109].

**Fig. 8:**
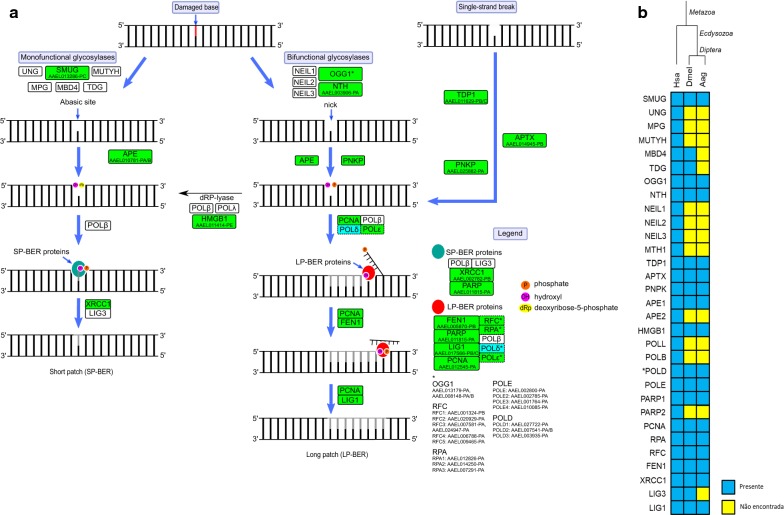
**a** Base excision repair (BER) repair in *Ae. aegypti*. Rectangles: solid line, protein; dashed, protein complex; green, identified; cyan, partially identified; white not identified. **b** Heatmap of *H. sapiens* (Hsa), *D. melanogaster* (Dmel) and *Ae. aegypti* (Aag) proteins. Protein codes are provided in Additional file 2: Table S8. *Subunits not identified: Aag-POLD4 and Dmel-POLD4

The mechanism of BER involves five major steps, and starts with the recognition and excision of the damaged base by a DNA glycosylase, that can be mono- or bifunctional and cleaves the N-glycosidic bond generating an AP site [73, 110, 111]. Humans possess eleven glycosylases: uracil DNA N-glycosylase (UNG); thymine DNA glycosylase (TDG); single-strand-selective monofunctional uracil DNA glycosylase (SMUG); methyl-CpG-binding domain 4 (MBD4); 3-methylpurine glycosylase (MPG); 8-oxoguanine DNA glycosylase (OGG); MutY homolog DNA glycosylase (MUTY); endonuclease III-like (NTH); endonuclease VIII-like 1 (NEIL1); endonuclease VIII-like 2 (NEIL2); and endonuclease VIII-3 (NEIL3). In *Ae. aegypti* there are only three DNA glycosylases: the monofunctional SMUG (AAEL013286-PC); the bifunctional OGG (AAEL013179-PA, AAEL008148-PA/B); and NTH (AAEL003906-PA) (Fig. 8). Comparing with other organisms, the monofunctional glycosylases UNG, MUTY, MPG are absent in the Diptera while MBD4 is present only in some species of this group such as *D. melanogaster*; and TDG is absent in mosquitoes [15]. The absence of UNG (the major uracil DNA glycosylase) in *D melanogaster*, and the downregulation of deoxyuracil triphosphatase (dUTPase) has already been correlated with high levels of uracil incorporation in larvae DNA. The higher levels of uracil-containing DNA are well tolerated in larval stages but corrected during development [15, 112]. In fact, *D. melanogaster* encodes a protein, denominated uracil-DNA degrading factor (UDE), present in holometabolous insects, which can degrade uracil-containing DNA [113]. The UDE protein is also found in *Ae. aegypti* (AAEL003864-PA), indicating that this mosquito may deal with uracil-containing DNA in the same way as *D. melanogaster*. The glycosylases OGG1, MUTY and the hydrolase MTH1 (named MutT in bacteria) are involved in the repair of the major oxidative lesion 7,8-dihydro-8-oxoguanine (8-oxoG) [114]. MTH1 catalyzes the hydrolysis of 8-oxo-dGTP to avoid its incorporation in DNA [115]. OGG1 is responsible for the removal of 8-oxoG residues from the DNA initiating the repair that will restore the G:C base pair [116]. If the 8oxoG:C bypass the excision by OGG1 and the DNA replication occurs, one of the DNA copies will have 8-oxoG:A pair. It is recognized by MUTY, that also removes the mismatched adenine [117]. In *Ae. aegypti* only an ortholog for OGG1 was identified. Otherwise, the 8-oxoG:A generated during DNA replication can be repaired by the MMR pathway [118] which, as discussed above, seems to be functional in this insect.

The second step is the action of the AP endonuclease (APE), which cleaves the DNA backbone at the 5′-end removing the remaining sugar-phosphate structure. When the damaged base is removed by a bifunctional glycosylase its lyase activity cleaves AP-site leaving an 3′α,β-unsaturated aldehyde (3′-PUA) or a phosphate group (3′-P), that are removed by APE and polynucleotide kinase 3′-phosphatase (PNKP), respectively [108, 119]. In the case of SSBs the DNA ends can be processed, to generate the necessary 3′- and 5′-termini, by aprataxin (APTX), tyrosyl-DNA phosphodiesterase 1 (TDP1) and PNKP [8]. Humans possess two APE, APE1 and APE2, but *Ae. aegypti* encodes only APE1 (AAEL010781-PA, AAEL010781-PB) and is lacking the APE2 ortholog that is also lacking in all dipterans [15]. Otherwise, PNKP (AAEL025882-PA), APTX (AAEL014945-PB-D) and TDP1 (AAEL011629-PB-C) are all present in *Ae. aegypti* (Fig. 8).

The next steps of BER can occur *via* two different pathways: short-patch (SP-BER) and long-patch (LP-BER). SP-BER proceeds when 3′-OH and 5′-dRP termini are present, in which DNA polymerase β (POL β) removes 5′dRP and inserts a new nucleotide, filling the gap. Then the complex of x-ray repair cross-complementing 1 (XRCC1) and DNA ligase 3 (LIG3) seals the nick [120]. The LP-BER occurs when 5′-terminal is not a Pol β substrate. In this pathway, between 2 and 10 nucleotides of the 3′-termini are displaced and removed from the DNA backbone and a new nucleotide chain is synthetized by any of the POL (β, δ or ε) complexed with PCNA and flap endonuclease 1 (Fen1). The final ligation step is performed by LIG1 [117].

As indicated above, POLβ and POLλ are members of Pol X family and both are lacking in *Ae. aegypti* and in the Diptera. It was already suggested that dipterans use only LP-BER due to the lack of POLβ [15]. Moreover, *Ae. aegypti* seems to have lost LIG3 (Fig. 8) while it is present in many dipterans (KEGG orthology group K10776), reinforcing the hypothesis that this specie uses only LP-BER pathway. The LP-BER polymerases POL δ and POL ε were found in *Ae. aegypti* (Fig. 8), but POL δ lacks the subunit 4 (discussed above). Humans possess two PARPs that participate in BER, PARP1 and PARP2. In *Ae. aegypti* only one ortholog of PARP (AAEL011815-PA) was identified (Fig. 8), which is more similar to PARP1. Although PARP1 and PARP2 have specific functions, both possess overlapping roles [121, 122]. Considering that PARP2 is absent is arthropods [25], it is possible that PARP1 is performing the functions of PARP2. The complete list of *Ae. aegypti* BER proteins is provided in Additional file 2: Table S8.

## Nucleotide excision repair

Nucleotide excision repair (NER) is a versatile pathway that repairs helix-distorting DNA lesions such as intra- and interstrand crosslinks and ultraviolet (UV) damages [123].

NER starts with damage recognition, which can occur *via* two sub-pathways: global genome repair (GG-NER) that detects lesions in all over the genome, and transcription-coupled repair (TC-NER) which recognizes damages in transcribed strand of active genes [124]. In GG-NER, detection occurs with the binding of the XPC complex (XPC, HR23B and CENT2) to the non-damaged strand, a process that is enhanced by the UV-DDB (XPE) complex (DDB1, DDB2 CUL4 and RBX1) [125].

**Fig. 9:**
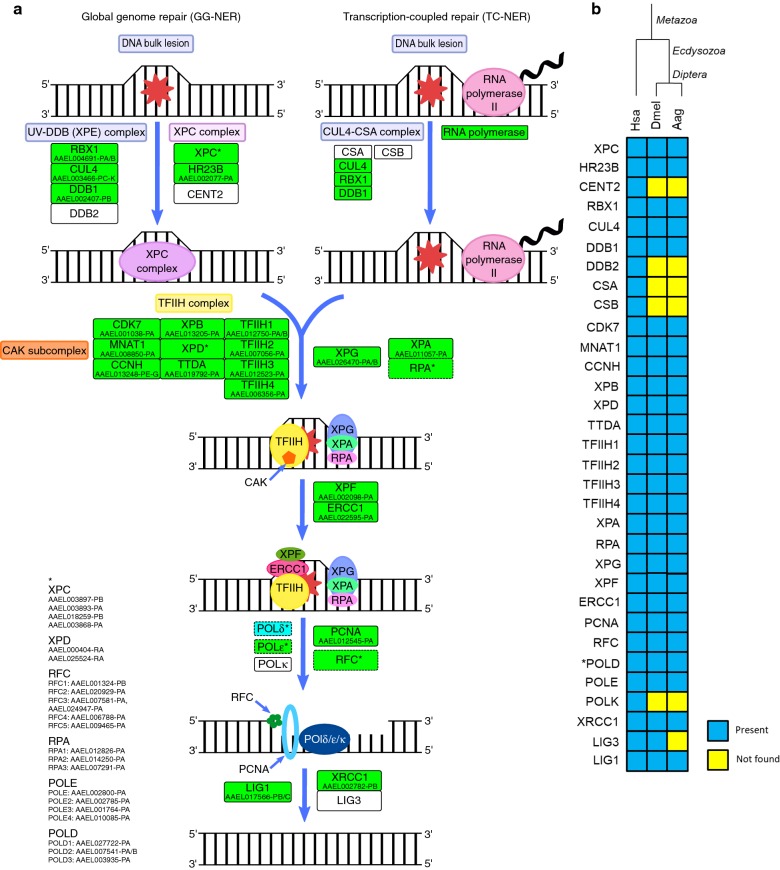
**a** Nucleotide excision repair (NER) repair in *Ae. aegypti*. Rectangles: solid line, protein; dashed, protein complex; green, identified; cyan, partially identified; white not identified. **b** Heatmap of *H. sapiens* (Hsa), *D. melanogaster* (Dmel) and *Ae. aegypti* (Aag) proteins. Protein codes are provided in Additional file 2: Table S9. *Subunits not identified: Aag-POLD4 and Dmel-POLD4

The two initial NER complexes emerged in early eukaryotes [25], except for the DDB2 that appears only in plants (KEGG orthology group K10140). The XPC complex (XPC - AAEL003897-PA/B, AAEL018259-PB, AAEL003868-PA; HR23B - AAEL002077-PA) is almost complete in *Ae. aegypti*, only CENT2 was not found (Fig. 9). Although CENT2 enhances the DNA-binding activity of XPC-HR23B, it is not essential for NER [126], thus the absence of this protein in *Ae. aegypti* may not interfere in GG-NER. Furthermore, the UV-DDB complex (DDB1 - AAEL002407-PB; CUL4A - AAEL003466-PC-K) was partially identified, lacking the DDB2 protein (Fig. 9). However, the XPC complex can recognize the damage in the absence of the UV-DDB complex, which indeed is necessary to keep the repair proteins around the lesion site [127]; so the lacking DDB2 probably will not avoid GG-NER but can decrease its efficiency (Fig. 9).

TC-NER repairs helix-distorting DNA lesions that block the RNA polymerase II such as inter- and intra-strand crosslinks generated by chemotherapeutics such as cisplatin, and UV damages such as cyclobutane pyrimidine dimers (CPDs) [128]. In this sub-pathway, the blockage of RNA polymerase II is the signal to recruit cockayne syndrome group A (CSA) and cockayne syndrome group B (CSB) proteins [123]. Both proteins were not identified in *Ae. aegypti* (Fig. 9), and seem to have been lost in all of the Diptera, otherwise the literature [15] states their origin in early eukaryotes. In humans, mutations in CSA and CSB proteins lead to cockayne syndrome, an autosomal recessive disease, characterized by microcephaly, photosensitivity, premature aging, short stature, learning and developmental delay [129]. Although mutations in these genes lead to a severe syndrome in humans, the TC-NER could not be identified in *D. melanogaster* [15] and is possible that DNA photolyases (discussed below) play a role in the repair of these UV lesions in these insects.

After damaged site recognition, both pathways require the same enzymatic machinery, and the following step is the recruitment of the ten subunits transcriptional factor IIH complex (TFIIH) [130].

The TFIIH helicases subunits XPB and XPD unwind DNA around the damage (~30 bp) generating a bubble, where the single strands are stabilized by XPA and RPA [73, 130, 131]. The following step is the dual incision around the damage, which is catalyzed by Xeroderma pigmentosum complementation group F (XPF)-DNA excision repair protein ERCC-1 (ERCC1) (5′) and XPG endonuclease (3′) [132]. The result is the removal of ~30 nucleotides, generating a gap that is filled by POL (δ, ε or κ), in cooperation with PCNA and RFC [133], and the remaining nick is sealed by XRCC1-LIG3 or LIG1 [134].

Arcas et al. [25] have already shown that NER central players originated in early eukaryotes, so is not surprising that these proteins are present in *Ae. aegypti*, except for LIG3 and POL δ, that lacks the subunit 4 (discussed above) (Fig. 9). The complete list of *Ae. aegypti* NER proteins is provided in Additional file 2: Table S9.

## Direct repair

In addition to the DNA repair pathways, discussed in this paper, the organisms also possess mechanisms that directly reverse the DNA damage. The DNA photolyases, the α-ketoglutarate-dependent dioxygenases (AlkB) and the O-6-methylguanine DNA methyltransferase (MGMT) are the main proteins involved in this type of DNA repair [135]. The complete list of *Ae. aegypti* direct repair proteins is provided in Additional file 2: Table S10.

### Photolyase repair

**Fig. 10:**
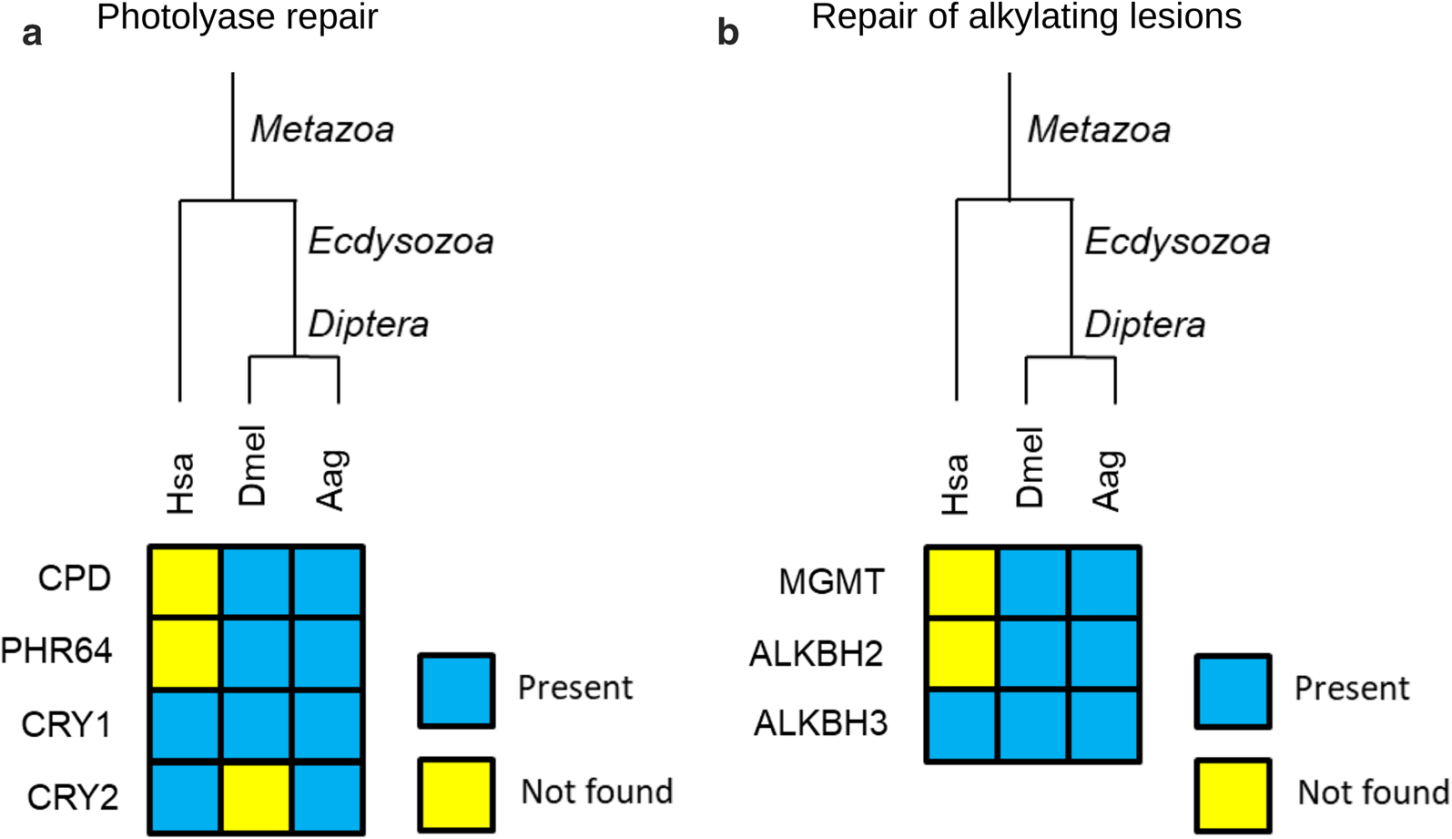
**a** Direct repair in *A*e*. aegypti*. Heatmaps of human (Hsa), *D. melanogaster* (Dmel) and *A*e*. aegypti* (Aag) proteins for photolyase repair and **b** alkylating lesions repair. Protein codes are provided in Additional file 2: Table S10

The photolyases are ancient flavoproteins activated by blue light that repair UV-induced DNA damages as cyclobutane pyrimidine dimer (CPD) and pyrimidine-pyrimidone (6-4) photoproduct [136]. Based on the substrate affinity, the photolyases are classified as CPD photolyase and (6-4) photolyase (PHR6-4). Photolyases are also ancestors of cryptochromes (CRY), a flavoprotein involved in circadian clock [137]. Although both are similar in sequence and crystal structure, CRY lacks the ability to repair UV-induced damage [138]. While humans only encode CRY, *D. melanogaster* possesses orthologs of the two types of photolyases plus CRY [139]. In *Ae. aegypti* it was possible to identify orthologs for CPD (AAEL001787-PA-E) and PHR6-4 (AAEL001175-PA), and two orthologs of CRY, CRY1 (AAEL004146-PA) and CRY2 (AAEL011967-PA) (Fig. 10a).

### Repair of alkylating lesions

Endogenous and exogenous alkylating agents can damage the genomic DNA by the generation of mutagenic and cytotoxic adducts. To deal with these lesions the cell encodes mechanisms to remove the alkylated base, such as DNA glycosylases and the direct repair enzymes MGMT and AlkB.

The MGMT repairs O-6-methylguanine, which is one of the most cytotoxic and mutagenic DNA lesions, due to the ability to pair with C and T during DNA replication [140]. The MGMT transfers the O-6-methyl group from guanine to its cysteine 145 [139]. The covalent bond between Cys145 and methyl group inactivates the MGMT, which is then degraded in the ubiquitin/proteasome pathway [141]. Although MGMT is a conserved protein that is also present in *D. melanogaster* [142, 143], an ortholog was not identified in *Ae. aegypti* (Fig. 10b), keeping unanswered how this mosquito deals with this alkylating lesion.

The DNA repair function of AlkB family dioxygenases was initially identified in *E. coli* AlkB, which removes the 1-methyladenine and 3-methylcytosine through an oxidative dealkylation reaction. Among the nine human AlkB orthologs only two (ALKBH2 and ALKBH3) possess the repair activity [144]. In *Ae. aegypti* orthologs for both ALKBH2 and ALKBH3 were not identified, which is also lacking in *D. melanogaster* (Fig. 10b).

Alkylating lesions can also be repaired by the DNA glycosylases from BER pathway, such as TDG, MBD4, MPG and SMUG [145]. Otherwise, *Ae. aegypti* only encodes ortholog for SMUG (Fig. 8). The absence of MGMT, ALKBH2, ALKBH3 and the DNA glycosylases raise questions about how this mosquito deals with alkylating DNA lesions.

## Conclusions

The bioinformatics analysis of this study helped identify orthologs of many key DDR proteins in *Ae. aegypti*, such as RAD51, RAD50, MRE11, NBN, KU80, KU70, LIG4, XLF, XPA, XPC, XPB, XPD, XPE, XPF, XPG, MSH2, MSH6, PMS2, MLH1 MutS, MutL, SMUG, OGG and NTH. Our analysis also identified a functional ortholog of human H2Ax (*Drosophila* H2Av) histone in *Ae. aegypti*. These findings indicate that the ATR and ATM signaling, DSB, HR, NHEJ, MMR, LP-BER and GG-NER repair pathways should be functional in this mosquito. Both insects showed similarities regarding the proteins not identified in *Ae. aegypti* (BRCA1 and its partners from the BRCA1-A complex, TP53BP1, PALB2, POLk, CSA, CSB and POLβ). It is relevant to stress that some unidentified proteins can be a result from real gene absences but also can represent a very divergent ortholog or a functional ortholog. In humans, almost all of them are essential and their lack affects DSB signaling, HR, GG-NER and SP-BER, raising questions about how these insects deal with DSB repair pathway choice and suggesting that both GG-NER and SP-BER could have been rewired or be absent. The differences between *Ae. aegypti* and *D. melanogaster* included seven proteins not reported in *D. melanogaster* that were found in *Ae. aegypti* (RNF168, RIF1, WRN, RAD54B, RMI1, DNAPKcs and ARTEMIS) and also other known six proteins in *Drosophila* that were not identified in *Ae. aegypti* (CTIP, DSS1, XRCC2, SLX4, XRCC4 and LIG3). Despite the lack of XRCC4 (important for NHEJ ligation step), NHEJ is functional in *Ae. aegypti*, since it was already used in the generation of genetically modified mosquitoes [18], suggesting a rewire of this pathway. This review provides an initial overview of DDR in *Ae. aegypti*. Understanding this system, especially the DSBs repair pathways, may help improve genomic manipulation and the establishment of transgenic mosquitoes.

## Supplementary information

**Additional file 1: Figure S1.** Reciprocal-blast based methodology workflow. Arrows labelled “a–e” represent BLASTp analysis where the queries are the arrow-base group proteins and the subject database is at the arrowhead. Forward BLASTp (arrow “a”) top 5 hits were considered if they have e-value < 10^−15^, forming “Hits” group. Reverse blasts (arrows “b–e”) top 2 hits were considered if they have e-value < 10^−15^.

**Additional file 2: Table S1.** ATR signaling protein codes. **Table S2.** DSB protein codes. **Table S3.** H2A codes. **Table S4.** HR protein codes. **Table S5.** NHEJ protein codes. **Table S6.** MMEJ protein codes. **Table S7.** MMR protein codes. **Table S8.** BER protein codes. **Table S9.** NER protein codes. **Table S10.** Direct repair protein codes.

## Data Availability

Not applicable.

## Abbreviations

DDR: DNA damage response, BER: base excision repair, NER: nucleotide excision repair, MMR: mismatch repair, HR: homologous recombination, NHEJ: non-homologous end joining, DSB: double-strand break
