# Supplementary figures and images for: DNA damage response and repair in perspective: Aedes aegypti, Drosophila melanogaster and Homo sapiens
Source: Parasit Vectors. 2019 Nov 11;12:533. doi: 10.1186/s13071-019-3792-1 (PMC6849265; doi:10.1186/s13071-019-3792-1)

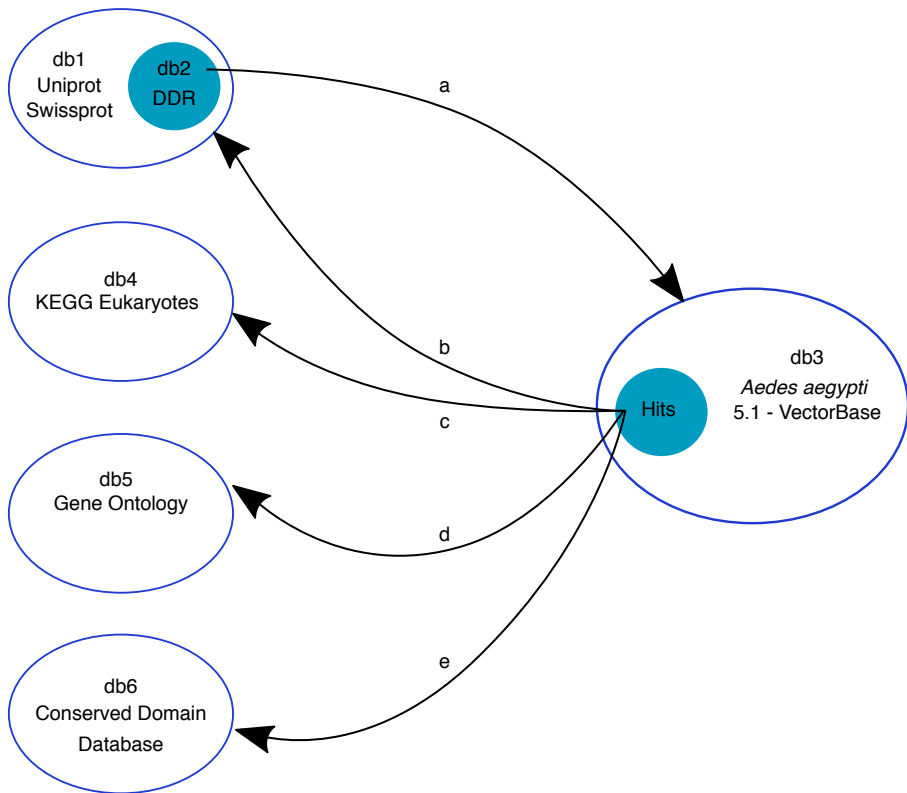

Supplement: Supplementary file 1 — Additional file 1: Figure S1. Reciprocal-blast based methodology workflow. Arrows labelled “a–e” represent BLASTp analysis where the queries are the arrow-base group proteins and the subject database is at the arrowhead. Forward BLASTp (arrow “a”) top 5 hits were considered if they have e-value < 10−15, forming “Hits” group. Reverse blasts (arrows “b–e”) top 2 hits were considered if they have e-value < 10−15. [file 13071_2019_3792_MOESM1_ESM.pdf]
